# Molecular strain typing of the yaws pathogen, *Treponema pallidum* subspecies *pertenue*

**DOI:** 10.1371/journal.pone.0203632

**Published:** 2018-09-12

**Authors:** Samantha S. Katz, Kai-Hua Chi, Eli Nachamkin, Damien Danavall, Fasihah Taleo, Jacob L. Kool, Kennedy Kwasi Addo, William Ampofo, Shirley V. Simpson, Tun Ye, Kingsley B. Asiedu, Ronald C. Ballard, Cheng Y. Chen, Allan Pillay

**Affiliations:** 1 Laboratory Reference and Research Branch, Division of STD Prevention, Centers for Disease Control and Prevention, Atlanta, Georgia, United States of America; 2 World Health Organization Country Office, Port Vila, Vanuatu; 3 Noguchi Memorial Institute for Medical Research, University of Ghana, Accra, Ghana; 4 Center for Global Health, Centers for Disease Control and Prevention, Atlanta, Georgia, United States of America; 5 Department of Control of Neglected Tropical Diseases, World Health Organization, Geneva, Switzerland; University of Helsinki, FINLAND

## Abstract

Yaws is a neglected tropical disease caused by the bacterium *Treponema pallidum* subspecies *pertenue*. The disease primarily affects children under 15 years of age living in low socioeconomic conditions in tropical areas. As a result of a renewed focus on the disease owing to a recent eradication effort initiated by the World Health Organization, we have evaluated a typing method, adapted from and based on the enhanced Centers for Disease Control and Prevention typing method for *T*. *pallidum* subsp. *pallidum*, for possible use in epidemiological studies. Thirty DNA samples from yaws cases in Vanuatu and Ghana, 11 DNA samples extracted from laboratory strains, and 3 published genomic sequences were fully typed by PCR/RFLP analysis of the *tpr E*, *G*, and *J* genes and by determining the number of 60-bp repeats within the *arp* gene. Subtyping was performed by sequencing a homonucleotide “G” tandem repeat immediately upstream of the *rpsA* gene and an 84-bp region of *tp0548*. A total of 22 complete strain types were identified; two strain types in clinical samples from Vanuatu (5*q*11/ak and 5*q*12/ak), nine strain types in clinical samples from Ghana (3*q*12/ah, 4*r*12/ah, 4*q*10/j, 4*q*11/ah, 4*q*12/ah, 4*q*12/v, 4*q*13/ah, 6*q*10/aj, and 9*q*10/ai), and twelve strain types in laboratory strains and published genomes (2*q*11/ae, 3*r*12/ad, 4*q*11/ad, 4*q*12/ad, 4*q*12/ag, 4*q*12/v, 5*r*12/ad, 6*r*12/x, 6*q*11/af, 10*q*9/r, 10*q*12/r, and 12*r*12/w). The *tpr* RFLP patterns and *arp* repeat sizes were subsequently verified by sequencing analysis of the respective PCR amplicons. This study demonstrates that the typing method for subsp. *pallidum* can be applied to subsp. *pertenue* strains and should prove useful for molecular epidemiological studies on yaws.

## Introduction

Yaws is an endemic treponematosis caused by the bacterium *Treponema pallidum* subsp. *pertenue* (TPE)[1]. It shares greater than 99.5% sequence identity with *T*. *pallidum* subsp. *pallidum* (TPA) and *T*. *pallidum* subsp. *endemicum* (TEN), the etiologic agents of venereal syphilis and bejel, respectively[2–4]. The three subspecies are indistinguishable on the basis of morphology and serology; however, differences in epidemiological characteristics and clinical presentation are apparent[5]. Though syphilis is usually acquired through sexual contact, yaws is commonly spread through skin-to-skin contact and bejel through contaminated eating utensils. Yaws and bejel are common among children under 15 years old living in low socioeconomic conditions in tropical (yaws) or arid (bejel) climates. The characteristic feature of yaws during the primary infectious stage is a solitary papilloma or ulcer on the lower limb or foot. If untreated, secondary manifestations appear which include ulcers, papillomata and/or papules, squamous macules, and palmar and plantar lesions. This may rarely be accompanied by periostitis of the long bones (saber shin) and fingers (polydactylitis). Late, non-infectious manifestations, although rare these days, result in destructive lesions of skin and bone in up to 10–20% of untreated individuals.

The World Health Organization (WHO) and United Nations International Children’s Fund (UNICEF) conducted a yaws eradication campaign in the 1950s and 1960s; however, the disease soon re-emerged and has continued to spread in parts of Africa, Asia, Latin America, and the Western Pacific[1,5]. In 2012, the WHO launched a new eradication effort to eliminate yaws by the year 2020, as a result of the demonstration that single-dose azithromycin was as effective as parenteral benzathine benzylpenicillin for yaws treatment[6]. However, despite many studies to determine the prevalence of the disease in various regions and advances in diagnostic methods[7–11], only one recent study has been published describing strain typing for TPE strains[12]. The authors of that study sought to develop a three-gene multilocus sequence typing (MLST) scheme for TPE, and three main strain types were identified among 190+ lesion swabs. We hypothesized that additional markers may be useful for molecular distinction of TPE strains.

For this work we adapted the Centers for Disease Control and Prevention (CDC) typing method for TPA strains to characterize TPE in clinical samples collected prior to mass drug administration (MDA) campaigns in Vanuatu and Ghana. The CDC typing method combines analysis of the number of 60-bp repeats within the acidic repeat protein (*arp*) gene with restriction fragment length polymorphism (RFLP) of the *T*. *pallidum* repeat (*tpr*) *E*, *G*, and *J* genes, despite in TPE the regions of *tpr G* and *J* used for RFLP are nearly identical and are thus expected to provide less discriminatory power than for TPA[13, 14]. The method used here also includes a subtyping component used for enhanced CDC typing which determines a sequence within the *tp0548* locus for greater discrimination[15]. In addition, we evaluated a separate subtyping component which relies on sequence quantification of a variable number of guanine repeats in a guanine homonucleotide run immediately upstream of the *rpsA* (*tp0279*) locus. Finally, sequencing analysis was used to verify observed *tpr* RFLP patterns and *arp* repeat sizes and sequences.

## Materials and methods

### Sample collection and DNA isolation

These studies were previously approved by the Ministries of Health in Vanuatu and Ghana and were approved at CDC under project determination # 7103 and 7162.

Cutaneous swabs were collected from clinically-suspected yaws lesions and stored in AssayAssure (Sierra Molecular, Incline Village, NV) medium prior to DNA extraction using the ChargeSwitch gDNA Mini Tissue Kit (Invitrogen, Carlsbad, CA). A total of 27 samples from suspected yaws patients on Tanna Island, Vanuatu[8] and 32 samples from the Abamkrom sub-municipality of Ghana that tested positive by PCR for TPE, as part of WHO yaws pilot studies, were included in the initial analysis. Following molecular testing of the DNA, full strain typing results for all targets examined were obtained only from 14 specimens from Vanuatu and 16 from Ghana, and thus only these data are presented here. Parental or guardian written and children’s verbal consent was given prior to examination of subjects. In addition, DNA from 11 TPE laboratory strains that were originally isolated from patients in Ghana (CDC 2575 and Ghana 051, isolated in 1980 and 1988, respectively)[16], Indonesia (K286, K319, K326, K344, K347, K348, K363, and K403, isolated in 1988)[17], and the Republic of Congo (Brazzaville, isolated in 1960)[16] were extracted from rabbit testicular extracts frozen in glycerol. DNA was extracted from the Nichols, Street 14, and JV-1 TPA strains grown in rabbit testes for use as controls[18]. 100 μL of each rabbit testicular sample was extracted using the ChargeSwitch gDNA Mini Tissue Kit and DNA was eluted in 100 μL elution buffer.

### Sequences analyzed for in silico analysis

Previously published whole-genome sequencing data from three TPE strains were used for *in silico* analysis and strain typing. These include Samoa D (isolated in Western Samoa in 1953), Gauthier (isolated in Nigeria in 1963), and CDC2 (isolated in Ghana in 1980) available under accession numbers CP002374-CP002376[19].

### PCR amplification of arp, tpr, tp0548, and rpsA genes for molecular typing

Strain typing was based on the CDC typing method for TPA[13]. The sequences of primers used for molecular analysis and amplicon sequencing are listed in **Table 1**. Briefly, a nested PCR was used for amplification of the *tpr E* (*tp0313*), *tpr G* (*tp0317*), and *tpr J* (*tp0621*) genes. Fifteen to 20 μL of DNA was used in a 50 μL reaction for the outer PCR with 2.6 units of Expand Taq polymerase (Roche Diagnostics, Indianapolis, IN, USA), 1x PCR buffer, 200 nM dNTPs, and 0.2 μM each of primer B1 and A2. Reaction conditions were as follows: 94°C for 5 min, 35 cycles of 94°C for 30 sec, 60°C for 1 min, and 68°C for 2 min 30 sec, followed by a final extension at 68°C for 15 min. Two μL of the outer PCR product was used for the nested reaction using the following conditions using primers IP6 and IP7: 94°C for 5 min, 40 cycles of 94°C for 30 sec, 59°C for 1 min, and 68°C for 2 min, followed by a final extension at 68°C for 15 min.

**Table 1:** Primers used for molecular testing.

Primer	Sequence
Typing Primers	
ARP N1	5’–ATCTTTGCCGTCCCGTGTGC– 3’
ARP N2	5’–CCGAGTGGGATGGCTGCTTC– 3’
A2	5’–CTACCAGGAGAGGGTGACGC– 3’
B1	5’–ACTGGCTCTGCCACACTTGA– 3’
IP6	5’–CAGGTTTTGCCGTTAAGC– 3’
IP7	5’–AATCAAGGGAGAATACCGTC– 3’
TP0548 FP2	5’–GGTCCCTATGATATCGTGTTCG– 3’
TP0548 RP2	5’–GTCATGGATCTGCGAGTGG– 3’
220I	5’–GCGCCCCAGACCCGCTCT– 3’
220J	5’–GAGCGATGATCACGGTCCCCAT– 3’
Primers to Amplify Full-Length *tpr* Genes	
E1	5’–CAGGATTTTCCGGTTCATTG– 3’
E2	5’–TCACGCGTTTAATGTTCTGC– 3’
E3	5’–AACCGCTTTTGAGCGTGTTG– 3’
E4	5’–CGGTGTTTGGCCGGTTATTC– 3’
G9.89	5’–TTGCACTTCGCGTTGTTCTCC– 3’
G10.3026	5’–TGTGGGTGTGCTTTGACACCA– 3’
G6.232	5’–CTGCGGCCTGTCGCTCTTAG– 3’
TPRG8	5’–GGACAGTGTGTGGATTCTTC– 3’
J2	5’–CGGTGATTGCAGCTCGGAGT– 3’
J3	5’–AAAGGACAGGGCCGTTGAGC– 3’
TPRJ9Fl	5’–GAAAAGAAGGGTGAGGGGGCTA– 3’
TPRJ11	5’–CCTCGGCGGGTGTGGGTGTG– 3’
Sequencing Primers	
220I	5’–GAGCGATGATCACGGTCCCCAT– 3’
495F	5’–CGCTTCTCCTTCGCCCTC– 3’
120R	5’–GCTTAAGGAATCCGGCAAAGT– 3’
458R	5’–CGCCTCTACCTTCCCCTTGC– 3’
2124F	5’–CTGTGCACAGCTGCGTGCTGG– 3’

RFLP analysis was performed on the nested *tpr* PCR product by digesting 6 μL of the DNA in a 10 μL volume with 1x reaction buffer, 1x bovine serum albumin (BSA), and 2 units of the *Mse* I enzyme (New England Biolabs, Ipswich, MA, USA). The reaction was carried out at 37°C for 2 h, followed by heat-denaturation of the enzyme at 65°C for 20 min. Samples were subsequently run on an Agilent Bioanalyzer 2100 (Santa Clara, CA, USA).

The 60-bp acidic repeat region of the *arp* (*tp0433*) gene was amplified using primers ARP N1 and ARP N2[15, 20]. A 50 μL reaction mix contained 10 to 15 μL of DNA, 1.75 units of Expand Taq polymerase, 1x PCR buffer, 200 nM dNTPs, and 0.2 μM each of primers ARP N1 and ARP N2. PCR amplification was done using touchdown conditions as follows: 94°C for 2 min, then 13 cycles of 94°C for 30 sec, 74°C– 1°C/cycle for 45 sec, and 72°C for 1 min 30 sec, then 25 cycles of 94°C for 30 sec, 64°C for 45 sec, and 72°C for 1 min 30 sec, followed by a final extension at 72°C for 10 min. Amplicons were subsequently run on an Agilent Bioanalyzer. Repeat numbers were determined based on comparison with the Nichols TPA strain, which has 14 of the 60-bp repeats within *arp*[13].

A portion of the *tp0548* locus (bp 131–215) was amplified as described previously with minor modifications[21]. A 50 μL reaction mix contained 5 μL of DNA, 2.5 units of AmpliTaq Gold polymerase (Life Technologies), 1x PCR buffer, 1.5 μM MgCl_2_, 200 nM dNTPs, and 0.6 μM each of primers TP0548 FP2 and TP0548 RP2. Reaction conditions were as follows: 95°C for 2 min, then 40 cycles of 95°C for 1 min, 60°C for 2 min, and 72°C for 1 min, followed by a final extension at 72°C for 10 min. Samples were subsequently run on an Agilent Bioanalyzer. The PCR product was then purified using the QIAquick PCR Purification Kit (Qiagen Sciences Inc., Germantown, MD, USA) and prepared for cycle sequencing.

A homonucleotide tandem repeat region immediately upstream of the coding region of the *rpsA* target locus was amplified as described previously[15]. A 50 μL reaction mix contained 10 to 15 μL of DNA, 2.5 units of AmpliTaq Gold polymerase, 1x PCR buffer, 1.5 μM MgCl_2_, 200 nM dNTPs, and 0.2 μM each of primers 220I and 220J. Reaction conditions were as follows: 94°C for 2 min, then 45 cycles of 94°C for 15 sec, 59°C for 30 sec, and 72°C for 30 sec, followed by a final extension at 72°C for 5 min. Samples were subsequently run on an Agilent Bioanalyzer. The PCR product was then purified using the QIAquick PCR Purification Kit and prepared for cycle sequencing.

### PCR amplification to verify the typing method

To verify the RFLP patterns observed following restriction digestion, we used a nested PCR to amplify the full-length *tpr* genes separately for sequencing analysis[14]. Two μL of DNA was amplified in a 50 μL reaction with 2.5 units of TaKaRa polymerase (TaKaRa Bio, Inc., Mountain View, CA, USA), 1x PCR buffer, 200 nM dNTPs, and 0.2 μM of primers E1 and E2 for *tpr E*, G9.89 and G10.3026 for *tpr G*, or J2 and J3 for *tpr J*. Reaction conditions were as follows: 94°C for 1 min, then 35 cycles of 94°C for 30 sec, 60°C for 1 min, and 68°C for 3 min 30 sec, followed by a final extension at 68°C for 10 min. Two μL of outer PCR product were used for the nested reaction, which was carried out using the same conditions but with primers E3 and E4 for *tpr E*, G6.232 and TPRG8 for *tpr G*, and TPRJ9Fl and TPRJ11 for *tpr J*. Samples were run on an Agilent Bioanalyzer and subsequently purified using the QIAquick PCR Purification Kit for amplicon sequencing.

To verify the number of 60-bp repeats as well as the sequence of each repeat within *arp*, we purified all *arp* amplicons using the QIAquick PCR Purification Kit.

### Cycle sequencing and sequencing analysis

The BigDye Terminator v3.1 Cycle Sequencing Kit (Life Technologies) was used to sequence the purified *arp* and *tpr E*, *G*, and *J* gene amplicons. The oligonucleotides used for sequencing are listed in **Table 1**. Briefly, approximately 150 ng of DNA was mixed with 1 μL of BigDye, 1.5 μL of sequencing buffer, and 4 pmol of sequencing primer in a 10 μL final volume. Primers ARP N1 and ARP N2 were used to sequence *arp* while primers A2, B1, IP6, IP7, 495F, 120R, 458R, and 2124F were used to sequence each of the *tpr* genes. Cycle sequencing was performed on a GeneAmp PCR System 9700 thermal cycler (Applied Biosystems, Foster City, CA, USA) with an initial denaturation of 96°C for 1 min followed by 25 cycles of 96°C for 10 sec, 50°C for 5 sec, and 60°C for 4 min. The *tp0548* and *rpsA* amplicons were similarly sequenced with the following modifications: 5 ng of DNA was used with 2 μL of BigDye and 3.2 pmol of primer TP0548 FP2 or TP0548 RP2 for *tp0548* or primer 220E or 220J for *rpsA* and with an initial denaturation of 96°C for 10 min. Products were purified using the BigDye XTerminator Purification Kit (Applied Biosystems) and run on an Applied Biosystems 3130xl Genetic Analyzer. All assemblies of sequenced amplicons, analyses of amplicons and published genomic sequences, and *in silico* digestion of *tpr* were performed using Geneious software (Biomatters Ltd., Auckland, New Zealand).

### Statistical methods

Associations between categorical variables were determined by using the chi-squared test. Results for which *P<0*.*05* were considered to be statistically significant.

## Results

### Tpr RFLP patterns

**Fig 1:**
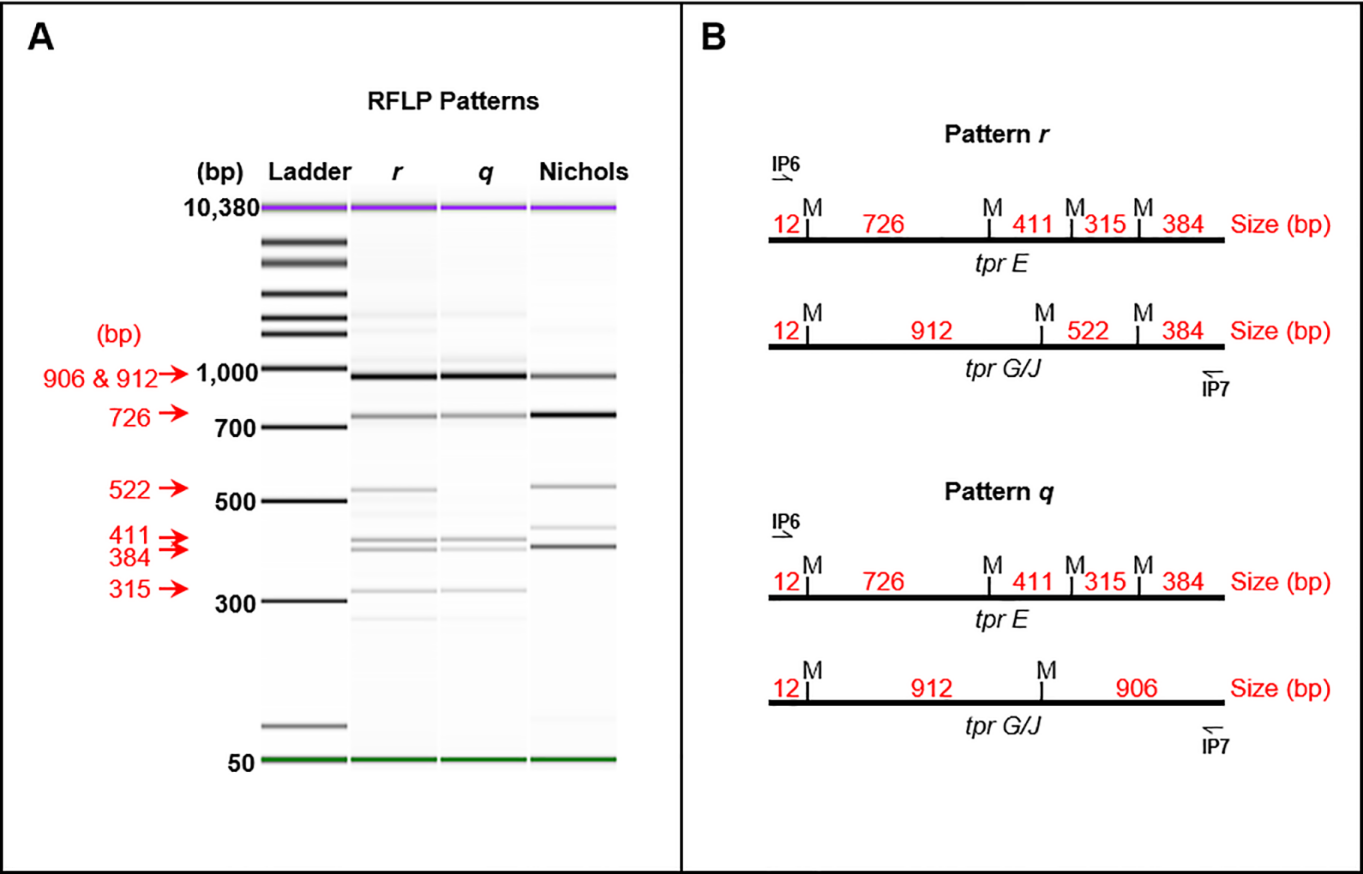
RFLP patterns of *T*. *pallidum* subsp. *pertenue*. (A) The *tpr E*, *G*, and *J* genes were amplified in a nested PCR to produce a mixed amplicon approximately 1830–1848 bp in length. Following PCR, amplicons were digested with the enzyme *Mse* I and analyzed on an Agilent Bioanalyzer. Two RFLP patterns (*q* and *r*) were observed among the clinical specimens and laboratory strains analyzed, including those for strains only partially typed (data not shown). The sizes of each fragment were confirmed through sequencing and are indicated in red. The Nichols TPA strain was used as a control for RFLP. The violet and green bands visible in each lane depict the Bioanalyzer kit’s internal control upper and lower bands, respectively. (B) Each of the *tpr E*, *G*, and *J* genes was amplified directly from the genome and sequenced. Regions of the products between the IP6 and IP7 primer annealing sequences were analyzed for *Mse* I restriction sites for both patterns. Approximate positions of each site (indicated as “M”) are shown. Sizes between each restriction site are listed in red.

Initially, 73 TPE strains were analyzed including 27 clinical specimens from Vanuatu, 32 clinical specimens from Ghana, 11 laboratory strains, and 3 published TPE genomes. Due to incomplete strain typing data at a single or multiple targets, a total of 44 TPE strains were fully typed including 14 clinical specimens from Vanuatu and 16 clinical specimens from Ghana; and 14 laboratory strains including 3 strains with whole genome sequences were strain typed. Following nested PCR amplification of the *tpr E*, *G*, and *J* genes and digestion with the enzyme *Mse* I two patterns, the previously identified pattern *q*[22] and new pattern *r*, were observed (**Fig 1A**). Six bands (approximately 320, 400, 420, 530, 750, and 950 bp in size) were observed in pattern *r* while five bands (approximately 320, 400, 420, 750, and 950 bp in size) were observed in pattern *q* (**Fig 1B**). Of these 44 strains, 100% (14/14) of the Vanuatu specimens and 94% (15/16) of the Ghana specimens were considered pattern *q* while 6% (1/16) of the Ghana specimens exhibited pattern *r*. Greater diversity of the two patterns was observed among the laboratory strains with 57% (8/14) considered pattern *q* and 43% (6/14) pattern *r*. A significant association was observed between RFLP pattern and geographic region (p = 0.0245), though a larger dataset may be necessary to understand if overrepresentation of the historic strains accounts for this.

To verify that the observed *tpr* RFLP patterns *q* and *r* are genetically relevant for molecular typing purposes, we sequenced the relevant regions of *tpr E*, *G*, and *J*. Each gene was amplified using gene-specific primers from several samples displaying either RFLP pattern. Following sequencing analysis of the regions between IP6/IP7 annealing sites we confirmed the presence and positions of *Mse* I restriction sites in each. In all *tpr E* samples that were examined, four restriction sites at positions 13, 739, 1150, and 1465 bp within the region were identified resulting in five fragments (**Fig 1B**). Specimens with RFLP pattern *r* displayed genetic variation between the *tpr G* and *J* genes. In some strains with pattern *r* the sequences of *tpr G* and *J* were identical with three restriction sites at positions 13, 922, and 1447 bp which resulted in four fragments approximately 12, 384, 522, and 912 bp in length in both genes. In other strains displaying pattern *r* the additional restriction site at position 1447 bp was not present in *tpr J*, though the resulting pattern was still visually indistinguishable from the other RFLP pattern *r* strains as the site was present within *tpr G*. This *Mse* I site at position 1447 bp was absent in both *tpr G* and *J* amplicons from RFLP pattern *q* specimens. Following sequencing analysis we performed *in silico* digestion using the enzyme *Mse* I on the relevant regions of the three genes from each sample. The digestion confirmed the sizes of each fragment produced in the *tpr* amplicon mixture as observed on the Agilent Bioanalyzer (**Fig 1A**).

### Analysis of 60-bp repeats within arp

**Fig 2:**
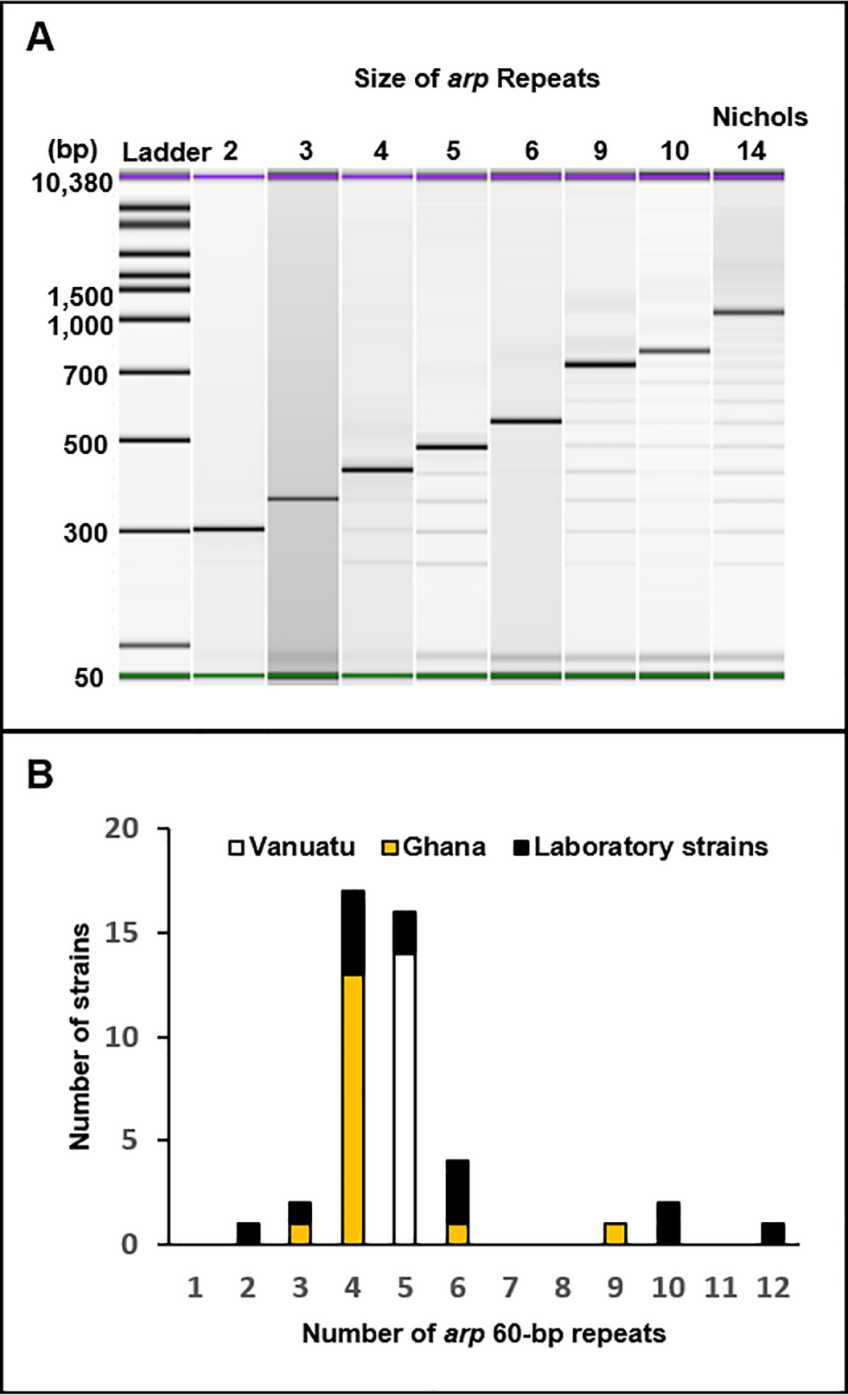
Analysis of the *arp* 60-bp repeat region. (A) A portion of the *arp* gene was amplified through PCR and run on an Agilent Bioanalyzer for each sample. Seven patterns containing 2, 3, 4, 5, 6, 9, and 10 repeats were identified among the samples tested in the lab, and an additional pattern (12 repeats) was seen following *in silico* analysis of one of the published genomes (not shown). The Nichols TPA strain, which contains 14 repeats, was used as a reference. The violet and green bands visible in each lane depict the Bioanalyzer kit’s internal control upper and lower bands, respectively. (B) Distribution of repeat sizes in clinical specimens from Vanuatu, Ghana clinical specimens, and among laboratory strains.

Eight different *arp* patterns (containing 2, 3, 4, 5, 6, 9, 10, or 12 60-bp repeats, respectively) were observed among the 44 strains fully typed, including the published genomes computationally analyzed (not shown) (**Fig 2A**). All 14 clinical specimens from Vanuatu had 5 *arp* repeats, including those only partially typed (data not shown), while the majority of clinical specimens from Ghana (13/16) had 4 repeats and a single strain each had 3, 6, and 9 repeats, respectively (**Fig 2B**). Of the Ghana strains partially subtyped, 4 60-bp repeats was also the only observable *arp* pattern (data not shown). Seven repeat patterns (containing 2, 3, 4, 5, 6, 10, or 12 60-bp repeats, respectively) were observed among the 14 laboratory strains and published genomes. Overall, *arp* repeat size was associated with geographic region (p<0.0001), with some *arp* patterns primarily localized in certain countries (e.g. Vanuatu).

In order to verify the number of 60-bp repeats within *arp* we sequenced the purified amplicons. Sequencing subsequently confirmed the number of repeats observed on the Agilent Bioanalyzer in each of the 44 samples fully strain typed. Additionally, for all samples the sequence within each 60-bp repeat was identical as expected based on previous studies by Harper *et al*.[23].

### Molecular subtyping by pattern determination within tp0548

**Fig 3:**
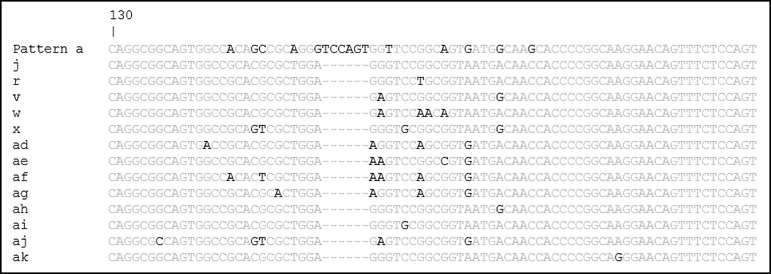
Alignment of the sequences using the variable region within *tp0548*. Amplicons containing a region of the *tp0548* locus were sequenced and analyzed for their genetic composition. The alignment compares the subtypes identified in this study to the Nichols TPA strain (pattern a). Thirteen subtypes, designated following nomenclature used for TPA strains and previously applied to TPE strains[12], were identified among the clinical specimens and laboratory strains, including those only partially strain typed.

A total of 13 subtyping patterns were obtained following sequencing of the *tp0548* locus or *in silico* analysis of published data (**Fig 3**), one of which matched a known TPA pattern and four of which were identified previously [12, 14]. Accordingly, *tp0548* subtyping pattern names for TPE strains were assigned according to the next available letter to be consistent with previous TPE designations. There was a significant association between *tp0548* subtype and geographic region (p<0.0001) with some subtypes only located in specimens from a single country. For example, a single subtype (ak) was observed in the Vanuatu clinical specimens, including among those only partially strain typed (data not shown). Five subtypes (j, v, ah, ai, and aj) were observed in the Ghana clinical specimens, including a single partially strain typed strain (which had pattern aj, not shown). Among them, subtype ah was the most common, being observed in 75% (12/16) of specimens fully strain typed. Eight subtypes (r, v, w, x, ad, ae, af, and ag) were observed in the laboratory strains with pattern ad (5/14) being the most frequent. Subtype v was observed in a single Ghana clinical strain, matching the pattern computationally determined for the TPE strain CDC2, which was isolated in Ghana in 1980.

### Molecular subtyping by tandem repeat determination within rpsA

Amplicons containing a portion of the *rpsA* locus were sequenced to determine the composition of a guanine homonucleotide tandem repeat upstream of the *rpsA* coding sequence. Five subtyping patterns were observed, containing 9, 10, 11, 12 or 13 repeats. Two subtypes were found in the Vanuatu samples with 71% (10/14) of the samples fully strain typed containing 11 repeats and 29% (4/14) containing 12 repeats, respectively. No additional subtypes were observed in the samples not fully strain typed, though a larger number of those (8/13) contained 12 repeats than 11 repeats (3/13) (data not shown). While 63% (10/16) of the Ghana specimens fully strain typed contained 12 tandem repeats, samples containing 10 (3/16 strains), 11 (2/16 strains), and 13 (1/16 strains) repeats were also found. Similar to the Vanuatu specimens, 12 repeats were more often seen among the Ghana samples not fully strain typed (5/16) than the other subtype identified (10 repeats, in a single strain) (data not shown). The most common *rpsA* subtyping pattern among the laboratory strains was 12 repeats as observed in 71% (10/14) of the samples. Other subtypes among the laboratory strains include 11 and 9 tandem repeats. Overall, there was a significant association between *rpsA* subtype and geographic region (p<0.0001).

We also sought to examine the stability and reproducibility of this locus. While a previous study noted inter- and intrastrain variability in homopolymeric tracts within *T*. *pallidum*[24], we observed stable sequences within the *rpsA* locus. Analysis of *rpsA* amplicons from the Street 14 TPA strain taken at three random rabbit passages over a 15-month period gave identical results (data not shown). This was consistent in five separate experiments using DNA from the Nichols, Street 14, and JV-1 TPA strains (i.e. consistently yielding 10, 9 and 9 repeats, respectively). Also, mixing three consecutive repeat sizes (9, 10, and 11) in different ratios followed by direct sequencing resulted in the observed repeat size being the same as the major repeat in the sample (data not shown).

### Strain types of TPE strains

Full strain typing results are shown in **Table 2**. Similar to the nomenclature used for enhanced CDC typing of TPA strains[21], strain types of TPE strains have been expressed as the number of 60-bp repeats (e.g. “5” in 5*q*12/ak), the *tpr E*, *G*, and *J* RFLP pattern (e.g. “*q*” in 5*q*12/ak), and the *tp0548* pattern (e.g. “ak” in 5*q*12/ak).When the number of tandem repeats within *rpsA* are included the designated strain type reflects the numerical count (e.g. “12” in 5*q*12/ak).

**Table 2:** Complete molecular strain types of *T*. *pallidum* subsp. *pertenue*.

	Number of specimens (% of region)						
	Clinical strains		Laboratory strains and *in silico* analysis of published sequences				
Strain type	Vanuatu n = 14	Ghana n = 16	Republic of Congo n = 1	Indonesia n = 8	Ghana n = 3	Nigeria n = 1	Western Samoa n = 1
2*q*11/ae	-	-	-	1 (12%)	-	-	-
3*r*12/ad	-	-	-	1 (12%)	-	-	-
3*q*12/ah	-	1 (6%)	-	-	-	-	-
4*r*12/ah	-	1 (6%)	-	-	-	-	-
4*q*10/j	-	1 (6%)	-	-	-	-	-
4*q*11/ad	-	-	-	1 (12%)	-	-	-
4*q*11/ah	-	2 (13%)	-	-	-	-	-
4*q*12/ad	-	-	-	1 (12%)	-	-	-
4*q*12/ag	-	-	-	1 (12%)	-	-	-
4*q*12/ah	-	7 (44%)	-	-	-	-	-
4*q*12/v	-	1 (6%)	-	-	1 (33%)	-	-
4*q*13/ah	-	1 (6%)	-	-	-	-	-
5*r*12/ad	-	-	-	2 (25%)	-	-	-
5*q*11/ak	10 (71%)	-	-	-	-	-	-
5*q*12/ak	4 (29%)	-	-	-	-	-	-
6*r*12/x	-	-	-	-	2 (67%)	-	-
6*q*10/aj	-	1 (6%)	-	-	-	-	-
6*q*11/af	-	-	-	1 (12%)	-	-	-
9*q*10/ai	-	1 (6%)	-	-	-	-	-
10*q*9/r	-	-	1 (100%)	-	-	-	-
10*q*12/r	-	-	-	-	-	1 (100%)	-
12*r*12/w	-	-	-	-	-	-	1 (100%)

**Fig 4:**
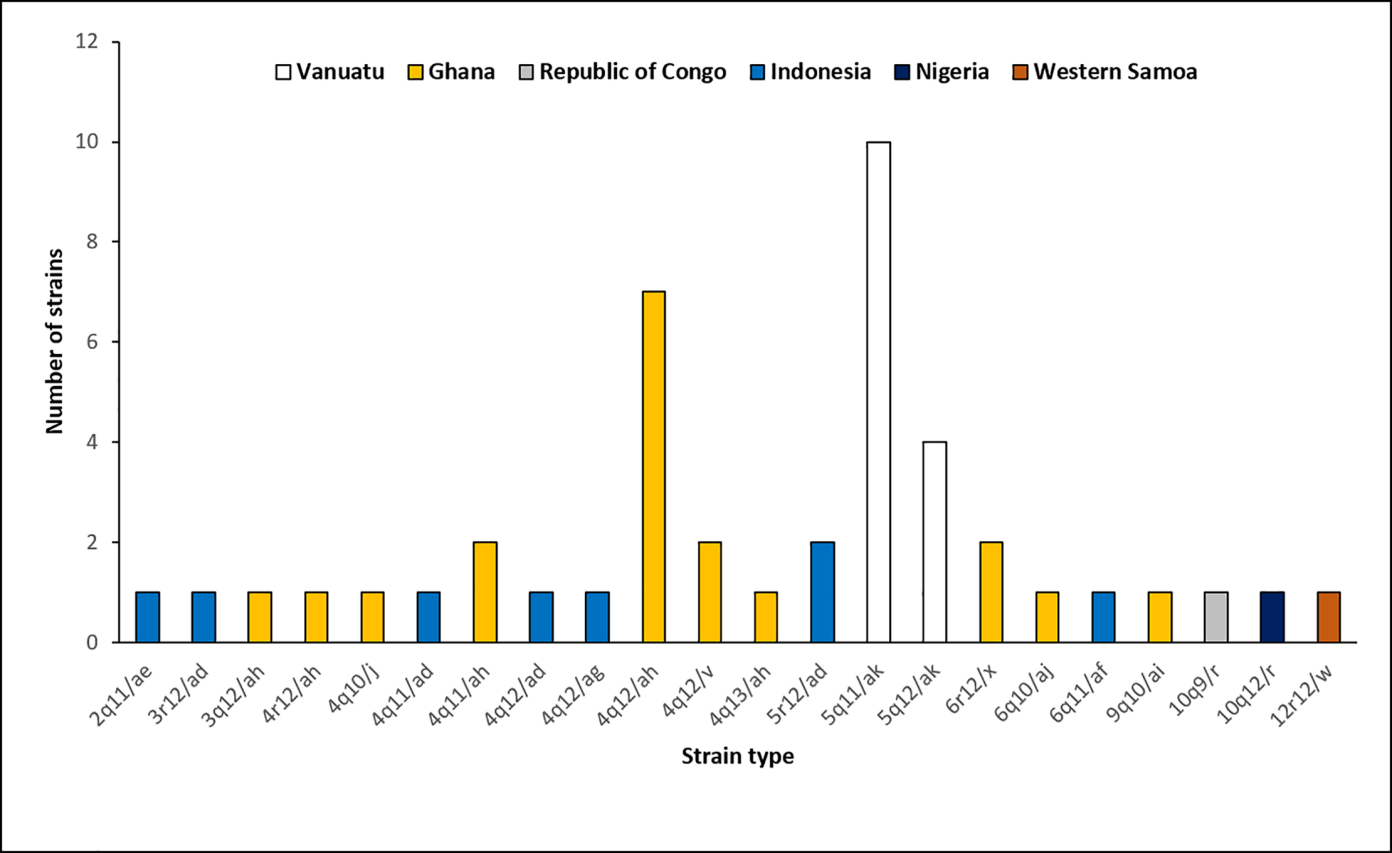
Distribution of TPE strains. Twenty-two strain TPE strain types were identified among the 44 specimens and genomic sequences analyzed. The overall distribution between each of the geographic locations is shown.

Using only the original target loci in the CDC molecular typing method consisting of the *tpr* RFLP and *arp* 60-bp repeat analysis, a total of 12 different types were identified among all samples fully strain typed. A total of 18 strain types were observed following the addition of the *rpsA* data to *tpr* and *arp*, and 17 strain types were observed instead with the addition of *tp0548* data to *tpr* and *arp*. A total of 22 strain types were identified when all 4-target loci were combined for typing (**Table 2**). The distribution of strain types among the different countries reflects the diversity of TPE strains in yaws-endemic regions (**Fig 4**).

Associations between RFLP pattern and *tp0548* subtype (p = 0.011) as well as *arp* repeat size with both *tp0548* subtype (p<0.0001) and *rpsA* subtype (p = 0.0075) were observed, while there was no association between RFLP pattern and *rpsA* pattern. Additionally an association between *tp0548* and *rpsA* subtypes (p = 0.0042) was observed, which indicates complementarity of these subtyping components when applied to TPE strains. Finally, there is an association between strain type and geographic region (p<0.0001), which appears evident from the presence of certain strain types in singular locations (e.g. 5*b*11/ak, which was found in high numbers only among the Vanuatu specimens).

## Discussion

The original TPA CDC typing method, as well as its variations[15, 21], has been successfully used in characterizing syphilis cases worldwide since the introduction of TPA typing two decades ago[13, 15, 20, 25–28]. Strain typing has subsequently proved a valuable tool in molecular epidemiologic studies during surveillance in specific regions, the emergence of macrolide resistance and during specific outbreaks[29–35]. While yaws is perhaps not as well-known as syphilis, it has become the focus of renewed interest as a result of the disease eradication campaign initiated by the WHO in 2012[1]. Recently, Godornes and colleagues applied a novel MLST scheme for TPE strains to 194 lesion samples from Papua New Guinea, and they found three predominant sequence types[12]. While their work presents a promising new method for molecular characterization of yaws strains, to our knowledge the scheme has not been applied to syphilis strains; therefore, it is more challenging for strain typing comparisons of yaws and syphilis. The goal of our present study was to evaluate the use of the CDC genotyping method for syphilis in molecular epidemiological studies of yaws.

In order to determine whether the typing system could be applied to TPE strains, we initially tested 73 specimens from PCR-confirmed yaws cases in Vanuatu and Ghana as well as laboratory strains originally isolated from Indonesia and parts of Africa. While a direct comparison of the typing method described here to the MLST scheme recently published[12] would provide a better picture of the utility of either approach, due to low specimen volumes or lack of DNA we were unable to address this in the present study. Four target loci were used for molecular typing, including the 60-bp repeat region within *arp*, *tpr* for RFLP analysis, a variable region within *tp0548*, and a homonucleotide tandem repeat within *rpsA*. The CDC typing method was fully applied to 44 TPE strains, including 41 tested in the lab and 3 computationally analyzed; however, several differences compared to TPA strains were noted.

First, the observed RFLP patterns differ from those found in TPA strains as genetic variations between the two subspecies exist at the *tpr* loci. In addition, because *tpr G* and *J* are identical in several TPE strains there is decreased discrimination using this genotyping component compared with application to TPA strains, as evidenced by the sheer number of RFLP patterns among syphilis strains while only two among the 44 yaws strains were revealed in this study. Next, sequencing analysis of *arp* amplicons revealed only the type II repeat motif was present among the TPE strains analyzed though in syphilitic strains there is more variability, as reported in the literature[18, 23]. Though the researchers in these studies concluded that diversity in *arp* correlated with transmission mode, further analysis of the significance of a singular motif in *arp* is beyond the scope of this paper and was not explored in the work presented here. Inclusion of the *tp0548* and *rpsA* subtyping methods allowed for greater discrimination of the yaws strains than the original CDC typing targets alone, similar to previous findings in syphilis[15, 21]. These findings support the inclusion of both subtyping targets for strain typing of TPE strains. Associations between the individual components of the typing scheme (including RFLP pattern with *tp0548* subtype, *arp* repeat size with *rpsA* and *tp0548*, and between the subtyping components) should be explored to understand if there is a genetic preference or if these findings simply reflect the genetic diversity of the strains examined here.

Of the 44 fully typed TPE strains, 22 strain types were identified using the 4-component system (**Table 2**). No strain type was observed in more than one country (**Fig 4**). We observed only two strain types among the Vanuatu specimens, which probably reflects infrequent introduction of new strains from the outside world into the rural communities of this remote island nation, while the genetic diversity observed in Ghana possibly reflects higher endemicity or transmission between communities. While the low sample size could have influenced these findings, we did observe a significant association between strain type and geographic region (p<0.0001) which appears to support these observations. Furthermore, each of the targets used for strain typing was associated with geographic region (p<0.05), highlighting their utility for distinction of TPE strains.

It should be noted that more researchers are moving away from complex typing schemes due to difficulties with amplification of long tracts of repetitive sequences and issues with reproducibility of RFLP patterns. Interestingly, in this study we initially found a “third” RFLP pattern that could not be confirmed upon sequencing analysis of the *tpr* genes examined. Inclusion of the roughly 80-nt region of *tp0548* for enhanced CDC typing greatly improves the discriminatory ability of strain typing compared with the original typing scheme, and researchers in the Czech Republic have identified further genetic variations in the locus for molecular distinction[21, 29]. However, phylogenetic analyses of these longer regions of *tp0548* indicate it is not useful for distinction of the TPA and TPE/TEN lineages[4]. Difficulties such as these have prompted exploration of sequence-based approaches and additional targets for yaws and syphilis studies. Though several studies have examined the utility of sequencing various targets for strain typing with promising findings[12, 36–38], the enhanced CDC typing remains a useful and highly discriminatory tool for molecular characterization of syphilis strains.

Overall, our data demonstrate that the CDC typing method for syphilis can be effectively applied to yaws strains. Application of the procedures described here to characterize TPE-specific DNA from confirmed yaws cases could prove useful in genetic characterization of the disease.
